# State of the art treatment of hepatitis B virus hepatocellular carcinoma and the role of hepatitis B surface antigen post‐liver transplantation and resection

**DOI:** 10.1111/liv.15124

**Published:** 2021-12-20

**Authors:** Peter Schemmer, Patrizia Burra, Rey‐Heng Hu, Christian M. Hüber, Carmelo Loinaz, Keigo Machida, Arndt Vogel, Didier Samuel

**Affiliations:** ^1^ General, Visceral and Transplant Surgery Department of Surgery Medical University of Graz Graz Austria; ^2^ Department of Surgery, Oncology, and Gastroenterology Padua University Hospital Padua Italy; ^3^ Department of Surgery National Taiwan University Hospital Taipei Taiwan; ^4^ Biotest AG Dreieich Germany; ^5^ Department of General and Digestive Surgery University Hospital 12 de Octubre Madrid Spain; ^6^ Keck Hospital of University of Southern California Los Angeles California USA; ^7^ Department of Gastroenterology, Hepatology and Endocrinology Medizinische Hochschule Hannover Hannover Germany; ^8^ Centre Hepatobiliaire University Hospital Paul Brousse University Paris‐Saclay and Inserm‐Paris Saclay Research Unit 1193 Villejuif France

**Keywords:** HBsAg, hepatitis B, hepatocellular carcinoma, resection, transplantation

## Abstract

Chronic hepatitis B virus (HBV) infection is the major aetiology of hepatocellular carcinoma (HCC). The optimal goal of therapy, hepatitis B surface antigen (HBsAg) loss and anti‐HBs production, is achieved rarely and HBsAg‐associated HCC risk is well recognized. Here we review the role of HBsAg in HCC, the link between HBsAg and HCC recurrence post‐liver transplantation or resection, and the implications for therapy. HBV‐associated carcinogenesis is a multifactorial process. The observation that HBV‐related HCC can occur in the absence of cirrhosis is compatible with a direct oncogenic effect of the virus, which may occur via multiple mechanisms, including those mediated by both mutated and unmutated HBsAg. HCC recurrence in HBsAg‐positive patients post‐liver transplantation has been reported in 10%‐15% of patients and is likely to be because of expansion of residual HCC tumour cell populations containing integrated HBV DNA, which expand and independently replicate HBV, leading to the recurrence of both HCC and HBV. The direct role of HBsAg in HCC recurrence post‐liver resection is less clear. Cirrhosis is the most important risk factor for HCC development, and precancerous cirrhotic liver remains after resection, with the potential to undergo malignant transformation regardless of the existence of HBV‐derived oncogenic drivers. The role of HBsAg in the development of HCC and its recurrence post‐surgical intervention has multiple implications for therapy and suggests a potential role for immunotherapy in the future management of HCC, in particular post‐liver transplantation. Use of hepatitis B immunoglobulins that target HBsAg directly, alongside immune‐oncology therapies, may be relevant in this setting.

**Abbreviations**
ALT: alanine aminotransferase
anti‐HBs: hepatitis B surface antibody
BCLC: Barcelona Clinic Liver Cancer
cccDNA: covalently closed circular DNA
CTCs: circulating tumour cells
DNA: deoxyribonucleic acid
HBeAg: hepatitis B ‘e’ antigen
HBIG: hepatitis B immunoglobulin
HBsAg: hepatitis B surface antigen
HBV: hepatitis B virus
HCC: hepatocellular carcinoma
HCV: hepatitis C virus
HDV: hepatitis delta virus
IO: immune‐oncology
LHB: large HBV surface protein
LT: liver transplantation
MHB: medium HBV surface protein
NAs: nucleos(t)ide analogues
SHB: small HBV surface protein
SVPs: subviral particles
TICs: tumour‐initiating stem‐like cells
TNM: tumour (T), node (N), and metastasis (M)

**Key points**

- Hepatocellular carcinoma (HCC) recurrence in hepatitis B surface antigen (HBsAg)‐positive patients post‐LT is likely because of expansion of residual HCC tumour cell populations containing integrated hepatitis B virus (HBV) DNA
- Precancerous cirrhotic liver remaining after resection has the potential to undergo malignant transformation regardless of the existence of HBV‐derived oncogenic drivers
- Quantitative HBsAg may be a useful tool to predict primary development and late recurrence of HCC post‐liver resection
- Role of HBsAg in the development of HCC and its recurrence post‐surgery has implications for therapy and suggests a potential role for immunotherapy in the management of HCC, in particular, post‐LT
- Use of hepatitis B immunoglobulins that target HBsAg directly, alongside immune‐oncology therapies, may be relevant in this setting

## 1 INTRODUCTION

Hepatocellular carcinoma (HCC) accounts for 90% of all primary liver cancers, making it a major global health problem.^1^ The aetiology of most cases of HCC, related deaths and disability‐adjusted life‐years is chronic hepatitis B virus (HBV) infection^2^; in 2015 there were 272 000 new cases of liver cancer attributable to HBV compared with 249 000 and 196 000 because of alcohol use and hepatitis C virus (HCV) infection, respectively.^2^ Approximately 3.5% of the world's population, more than 240 million individuals, are estimated to be living with chronic hepatitis B,^3^, ^4^ defined as hepatitis B surface antigen (HBsAg) positivity. Although vaccination programmes have reduced the number of new HBV infections drastically, many millions of people are at risk of complications, such as HCC, because of HBV infection acquired decades ago.

**FIGURE 1:**
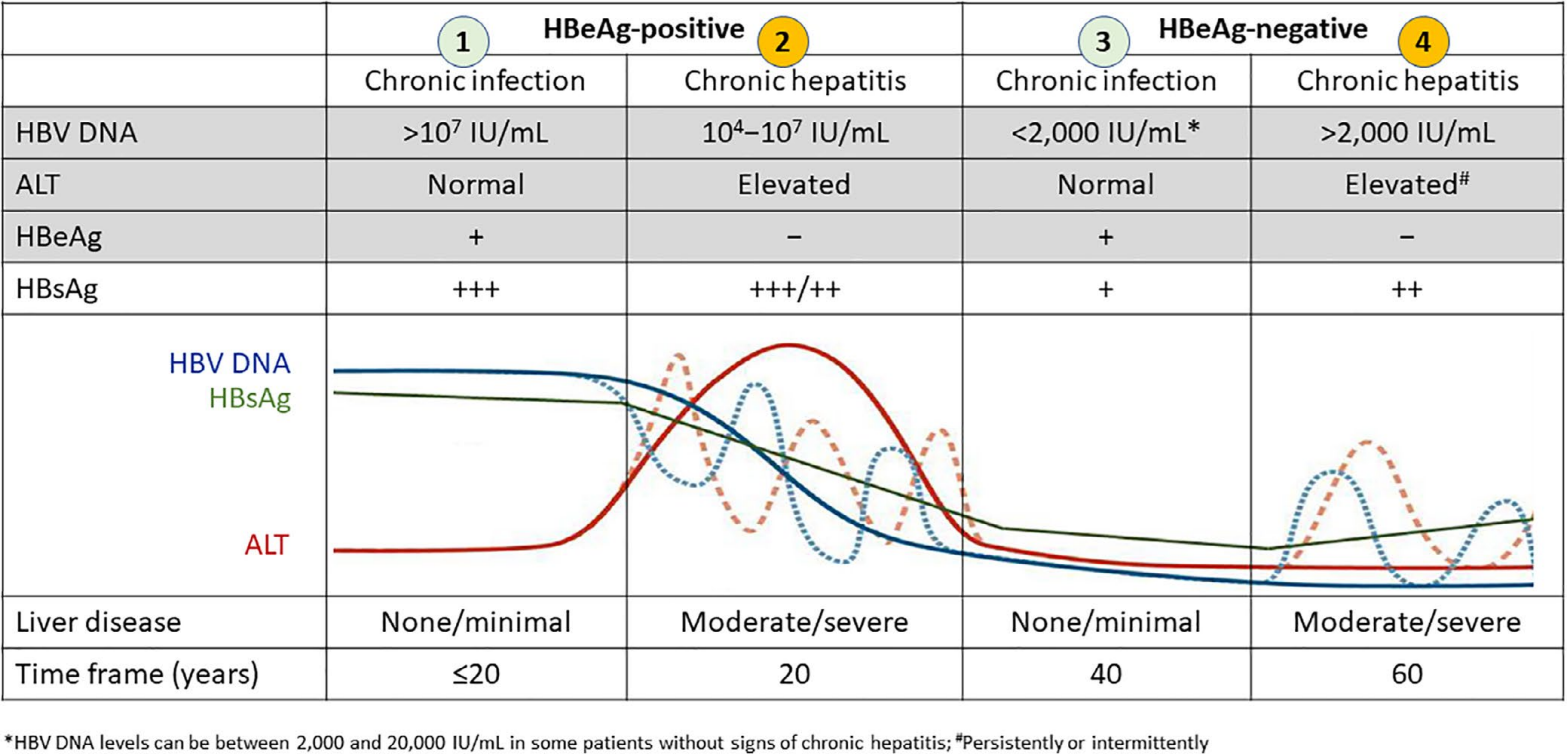
Natural history and phases of chronic HBV infection. ALT, alanine aminotransferase; HBeAg, hepatitis B ‘e’ antigen; HBsAg, hepatitis B surface antigen

The natural history of chronic HBV infection is variable and dependent on a complex interplay between the host immune response and the virus.^5^ Based on three clinical parameters (serum alanine aminotransferase [ALT] levels, serum HBV DNA levels, and hepatitis B ‘e’ antigen [HBeAg] levels), the disease is divided into four phases^3^, ^5^ (Figure 1): (1) HBeAg‐positive chronic infection (‘immune tolerant’); (2) HBeAg‐positive chronic hepatitis; (3) HBeAg‐negative chronic infection (‘inactive carrier’); (4) HBeAg‐negative chronic hepatitis. Patients in phases 2 and 4 are considered to have active disease and as such are eligible for treatment^3^ aimed at preventing disease progression and HCC development, primarily with long‐term potent nucleos(t)ide analogues (NAs) with a high barrier to resistance. Patients in all these phases are positive for HBsAg.^5^

The optimal goal of therapy, HBsAg loss and anti‐HBs production, is achieved rarely with NAs, and clinical cure is precluded by the persistence of HBV DNA within infected cells, either as covalently closed circular DNA (cccDNA) or integrated within the host genome. cccDNA serves as the transcription template for all viral RNAs, and together with integrated HBV DNA, is a major source of HBsAg.^6^ The continued transcription from cccDNA and integrated HBV DNA in the presence of NAs may explain the modest on‐therapy decreases in HBsAg levels despite undetectable levels of HBV DNA^5^ and highlights the imperfect correlation between these two markers of infection. In addition, HBsAg clearance requires an enhanced immune response from the host.

As well as formation of cccDNA to ‘hide’ HBV DNA,^7^, ^8^ the HBV virus evades host detection by dampening innate immune responses^7^, ^8^, ^9^, ^10^ and by producing subviral particles (SVPs) to divert the adaptive immune response and exhaust HBV antigen‐specific immune cells.^8^, ^10^ It is the eventual triggering of inflammatory and immune responses that leads to the accumulation of damage and progression of liver disease to fibrosis, cirrhosis, and ultimately HCC.

Multiple HBV viral factors have been implicated in the development of HCC,^11^ and HBsAg‐associated HCC risk has been recognized for decades. Patients remaining HBsAg positive are at much higher risk of developing HCC than their HBsAg‐negative counterparts,^12^, ^13^, ^14^ particularly if levels of HBV DNA are detectable,^15^ and have poorer outcomes.^16^ This HBsAg‐associated risk has been comprehensively reviewed recently.^16^, ^17^ The aim of our review is to identify the role of HBsAg in the natural history/development of HCC, the link between HBsAg and HCC recurrence or persistence post‐liver transplantation (LT) or resection, and the associated implications for therapeutic intervention.

## 2 METHODS

Oral presentations and discussions at a meeting convened by a multidisciplinary group of experts, including oncologists, surgical oncologists, transplant‐surgeons, molecular biologists, and hepatologists have been supplemented with a comprehensive literature search. The literature search was conducted on the PubMed database as follows: ((HCC) OR (hepatocellular carcinoma)) AND ((HBsAg) OR (hepatitis B surface antigen)) and was limited to studies performed in human subjects and reported in the English language over the last 5 years. This search conducted in June 2021 returned 538 publications for evaluation. After exclusion of references based on relevance, a total of 100 references were evaluated. These were supplemented with additional publications as appropriate.

## 3 RESULTS

### 3.1 HBsAg in the development of HCC

#### 3.1.1 HBsAg in the development of the primary HCC lesion

Hepatitis B virus‐associated carcinogenesis is a multifactorial process involving direct effects of viral proteins, indirect mechanisms through chronic inflammation and immune evasion, and the integration of HBV DNA.^18^ Although patients with HBsAg loss may still develop HCC and require surveillance,^17^ the risk is low and comparable in those who clear HBsAg spontaneously or following NA therapy,^19^ including in patients with HBsAg loss after finite NA therapy.^20^, ^21^ In young patients and those with HBsAg loss prior to the onset of cirrhosis the risk of HCC is minimal.^3^ Thus, the focus here is on patients remaining HBsAg positive.

**FIGURE 2:**
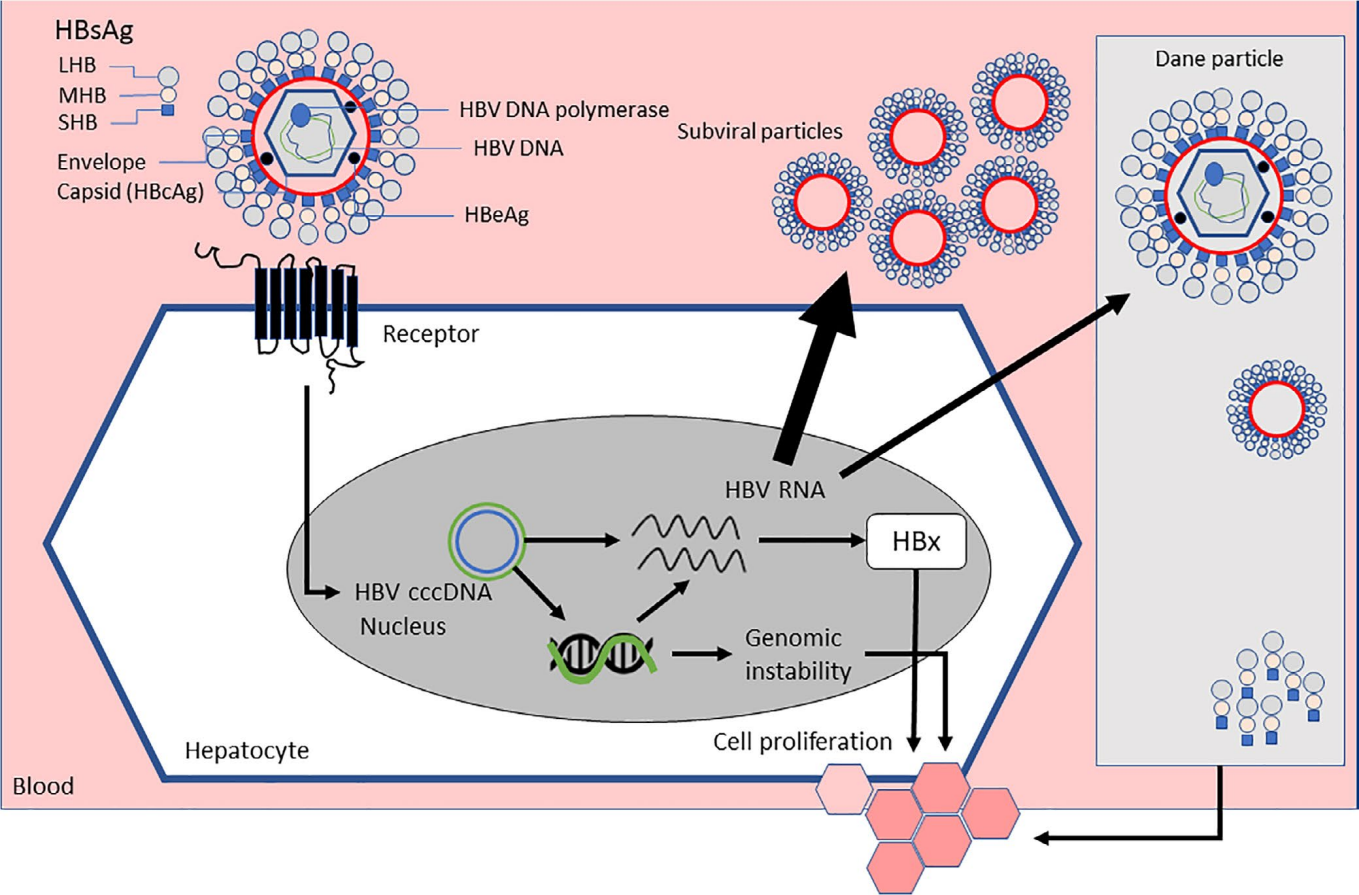
Potential role of HBsAg in development of HCC. Integration of HBV DNA into the host genome may result in genomic instability, which together with production of HBV proteins, may result in uncontrolled cell proliferation and tumour development. LHB, large HBV surface protein; MHB, medium HBV surface protein; SHB, small HBV surface protein; HBcAg, hepatitis B core antigen; HBeAg, hepatitis B ‘e’ antigen; cccDNA, covalently closed circular DNA

Although the majority of HBV‐related HCC develops in cirrhotic liver, unlike hepatitis C infection, some cases occur relatively early in the disease course, prior to development of cirrhosis or decompensated disease. This observation in individuals considered to have minimal liver damage is compatible with a direct oncogenic effect of HBV^22^ and has been cited as a reason for starting antiviral therapy in ‘immune tolerant’ patients,^23^ despite guidance to the contrary.^3^ The direct oncogenic effect of HBV may occur via multiple mechanisms. Integration of HBV DNA into the host genome may result in genomic instability, which together with production of HBV proteins, especially the regulatory protein HBx, may result in uncontrolled cell proliferation. Although the role of viral proteins remains unclear,^22^ Dane particles, HBsAg‐expressing SVPs and free HBsAg may contribute directly to tumour development^24^, ^25^, ^26^ (Figure 2), potentially via activation of the nuclear factor‐ΚB pathway.^27^

Hepatitis B surface antigen consists of large (LHB; pre‐S1), medium (MHB; pre‐S2), and small (SHB; S) HBV surface proteins, and mutations in the coding regions for these proteins have been shown to increase as HBV‐related disease progresses and to confer a high risk of HCC,^28^ leading to loss of genome integrity,^29^ with some found to be more common in patients with cirrhosis/HCC compared with those without.^30^ Recently, SHB has been shown to induce the epithelial‐mesenchymal transition process in HCC cells, significantly increasing their migratory and invasive ability as well as their metastatic potential.^31^ In the same study, in HCC sections from patients who underwent curative resection, expression of SHB in HCC tumours was positively correlated with a more aggressive phenotype, namely higher tumour number, loss of tumour encapsulation and a higher TNM stage, and reduced post‐resection survival.^31^ Mutations resulting in impaired HBsAg secretion and cellular proliferation,^29^ as well as calcium homeostasis and chromosome instability,^32^ and altered host gene expression,^33^ have been identified. As well as potentially having a direct role in carcinogenesis, these mutant HBsAg proteins may have a role to play in the ability of HBV to evade immune detection.

The direct oncogenic potential of HBsAg is supported by preclinical studies and suggests that unmutated HBsAg also has oncogenic potential. In an HBsAg transgenic mouse model, HBsAg accumulation in hepatocytes activated cancer stem cell markers to potentiate HCC development.^22^ Pre‐S1 has been shown to act as an oncoprotein and to play a key role in the appearance and self‐renewal of cancer stem cells during HCC development.^26^ Knockout of HBsAg in an HCC cell line resulted in decreased expression of HBsAg and significant attenuation of HCC proliferation in vitro as well as tumorigenicity in vivo.^34^ In the same study, overexpression of all three HBsAg surface proteins promoted proliferation and tumour formation.^34^ HBsAg levels may also lead to endoplasmic reticulum stress and a pre‐malignant phenotype.^35^ Together, these observations suggest that neutralising the effect of HBsAg could have a beneficial effect in HBV‐associated HCC.

As well as direct promotion of carcinogenesis, it is also thought that evasion from self‐immunity may be necessary for cancer growth. Given the observation that HBsAg carriers have a 25‐37‐fold increased risk of developing HCC compared with non‐infected people, HBsAg may have a function in immune evasion rather than being purely oncogenic,^18^ and may activate signalling pathways that link inflammation and tumour progression during chronic HBV infection.^36^ Although HBsAg has a tumour‐promoting effect, clearance of HBsAg reduces, but does not eliminate, the risk of HCC entirely,^16^, ^17^ and the risk persists particularly in cirrhotic patients, despite HBsAg clearance.

In the Taiwanese REVEAL cohort, relative risk of HCC was shown to be dependent on serum levels of HBsAg.^37^ Compared with levels <100 IU/mL, patients with levels of 100‐999 and ≥1000 IU/mL were found to be 2.8‐ and 4.1‐fold more likely to develop HCC, respectively,^37^ and in a further study,^12^ levels of HBsAg ≥ 1000 IU/mL conferred a 13.7‐fold increased HCC risk compared with lower levels. This HBsAg level‐dependent association was particularly apparent in patients with low HBV DNA,^12^, ^37^ indicating the importance of lowering HBsAg even in patients with low HBV DNA. Risk of HCC is also dependent on serum levels of HBV DNA, with cumulative incidence rates of HCC increasing from 1.3% to 14.9% with HBV DNA levels of ≤300 to ≥1 million copies/mL, respectively.^38^ HBsAg‐ and HBV DNA‐related increases were independent of HBeAg, serum ALT levels or cirrhosis. As a consequence, three cut‐offs for HBsAg (<100, 100‐999, ≥1000 IU/mL) and three for HBV DNA (<10^4^, 10^4^‐10^6^, ≥10^6^ copies/mL) have been included in risk scores for HBV‐related HCC.^37^ It remains unclear, whether the risk of HCC is higher because of increased liver inflammation in response to viral replication or to direct mechanisms. In a further recent analysis of data from the REVEAL cohort in patients remaining HBeAg‐positive throughout follow‐up, risk of HCC was found to be 3‐fold higher among those with declining HBsAg levels (low HBsAg levels at baseline or declining to <10 000 IU/mL) than in those with HBsAg levels persistently above 10 000 IU/mL without decline (*P* = .03).^39^

Although the increase in HCC is independent of cirrhosis, the majority of HBV‐related HCC is on the background of a cirrhotic liver. Indeed, cirrhosis is the most important risk factor, with the 5‐year cumulative risk of developing HCC being 0.6%‐2.4% in individuals with untreated chronic hepatitis B compared with 9.7%‐15.5% in those with compensated cirrhosis.^40^ The risk increases further with hepatic decompensation, with 3‐year rates of HBV‐related HCC in patients with decompensated disease being reported at 25%.^41^ Therefore, it is the combination of cirrhosis, in particular decompensated cirrhosis, and the serum levels of HBV DNA that particularly predispose to the development of HCC.

Coinfection with the hepatitis delta virus (HDV) is a key risk factor for the development of HCC. Approximately 15‐20 million people are infected with HDV, a subviral satellite that requires HBV for propagation.^42^ In coinfected patients, liver disease is more rapidly progressive, more likely to progress to HCC, and is associated with higher mortality than in those patients infected with only HBV.^42^, ^43^ The additional oncogenic capacity of HDV is not fully understood, although there is some evidence that small HDV antigens may have a direct role in carcinogenesis.^43^ In addition, HDV replication in hepatocyte nuclei results in dysregulation of pathways, increased oxidative stress and epigenetic changes that can lead to malignant transformation.^42^ Activation and coregulation of genes critically associated with DNA replication, damage, and repair are higher in HDV/HBV‐HCC hepatocytes than either non‐malignant or HBV‐HCC hepatocytes, suggesting that genetic instability may be an important mechanism in HDV/HBV‐related HCC and that, despite the dependence of HDV on HBV, distinct molecular mechanisms are involved.^44^

#### 3.1.2 Recurrence of HCC post‐LT

In early‐stage HCC, LT is a preferred treatment option.^1^ Although the prevalence of HBV‐related LT in Europe has stabilised over the last 30 years, HBV‐related HCC as an indication for LT has increased in contrast to HBV‐related decompensated liver disease.^45^ In addition, the incidence of patients undergoing LT with positive HBV DNA is increasing, and in patients with HBV‐related HCC as the indication for transplant, this is a predictor of reduced survival.^45^ In Asia, with the exception of Japan, HBV‐related HCC remains the most frequent indication for liver resection or LT. Although there is debate around whether the likelihood of HCC recurrence is higher in patients with HBV‐related disease compared with that of other aetiologies, there is little debate that HCC will recur in a proportion of patients.

Most patients will be HBsAg positive at the time of LT, and rates of HCC recurrence post‐LT in these patients have been reported to be around 10%‐15%.^46^ A proportion of patients with HCC recurrence will also be re‐infected with HBV. The rate of reinfection is higher than for other HBV‐related transplant indications,^47^ and both HBV reinfection and HCC recurrence are associated with poorer survival.^46^

**FIGURE 3:**
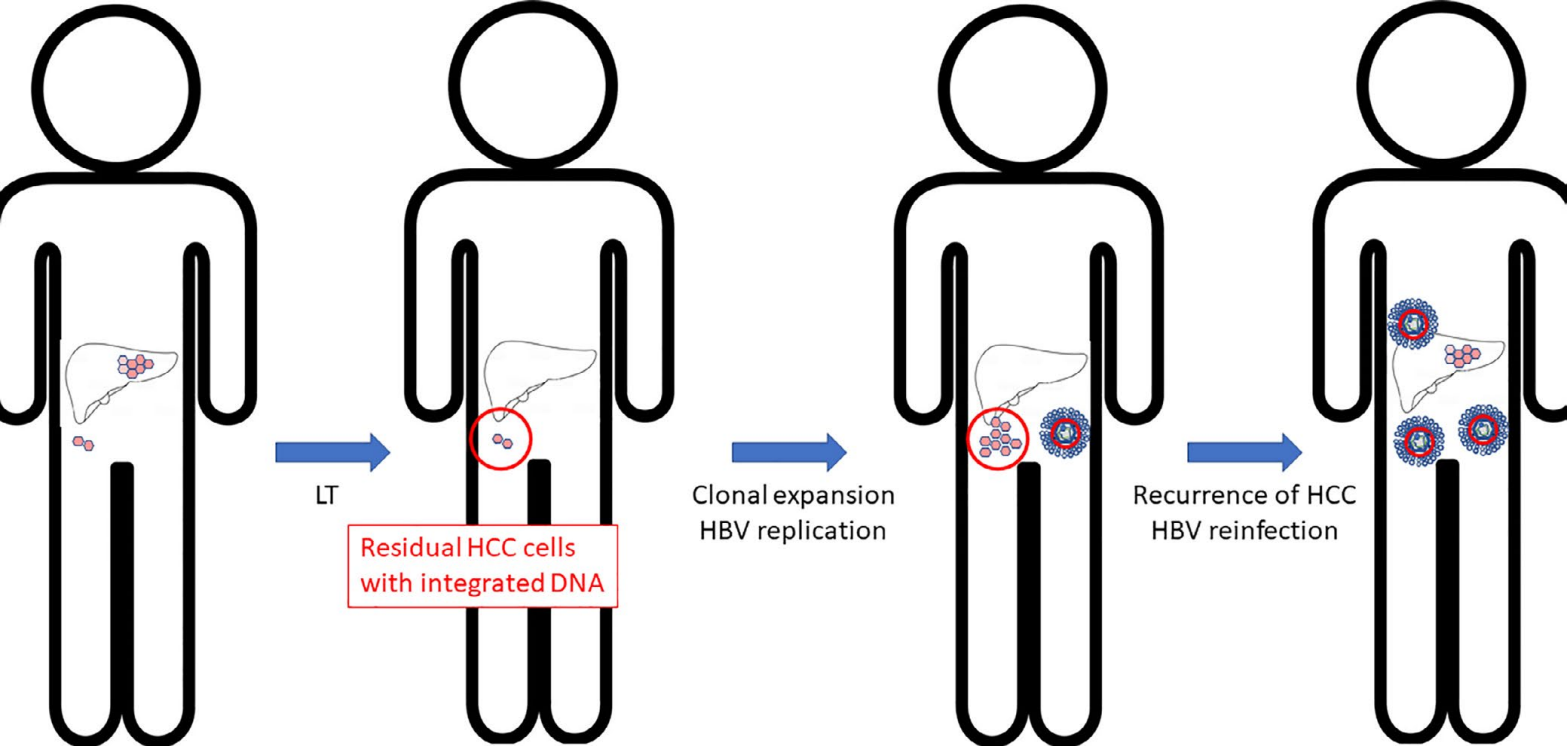
Potential cause of HCC recurrence and HBV reinfection post‐LT. Post‐LT residual HCC tumour cell populations containing integrated HBV DNA may expand and independently replicate HBV, leading to the recurrence of both HCC and HBV. LT, liver transplantation; HCC, hepatocellular carcinoma; HBV, hepatitis B virus

It is deemed critical to understand the cause of recurrence of HCC post‐LT. There are two hypotheses that can be put forward: firstly, that post‐LT residual HCC tumour cell populations, containing integrated HBV DNA, expand and independently replicate HBV, leading to the recurrence of both HCC and HBV^47^; secondly, that residual HBV RNA or HBsAg produced by other non‐tumour cells drive the reactivation of HBV, in turn driving the de novo recurrence of HCC. Data from a recent study in Korea (unpublished data) indicate that the first scenario may be more likely (Figure 3), since HBV DNA positivity was not linked to HBV reactivation, whereas recurrence of HCC was an independent risk factor for HBV reinfection. These data are further supported by observations that patients who become HBV DNA and HBsAg negative after removal of the primary tumour can have a recurrence of HCC expressing HBV DNA,^47^ indicating that the recurrence of tumour cells alone could drive the recurrence of HBV. In addition, low‐level oncogenic HBV variants persist post‐LT despite potent anti‐HBV prophylaxis and could lead to de novo carcinogenesis, as described earlier.

The immunosuppression required to minimize graft rejection in patients undergoing LT is also likely to contribute to HCC recurrence post‐LT. Tumour progression is more rapid and aggressive in immunosuppressed patients post‐LT,^48^ with the degree of immunosuppression having a negative impact on survival. Corticosteroids used in post‐LT immunosuppression protocols stimulate viral replication through binding to the glucocorticoid responsive enhancer region of the HBV genome. As a consequence, it has been proposed that these drugs should be removed from immunosuppressive regimens as soon as possible to reduce the likelihood of HBV reinfection and HCC recurrence.^49^

#### 3.1.3 Recurrence of HCC post‐liver resection

**FIGURE 4:**
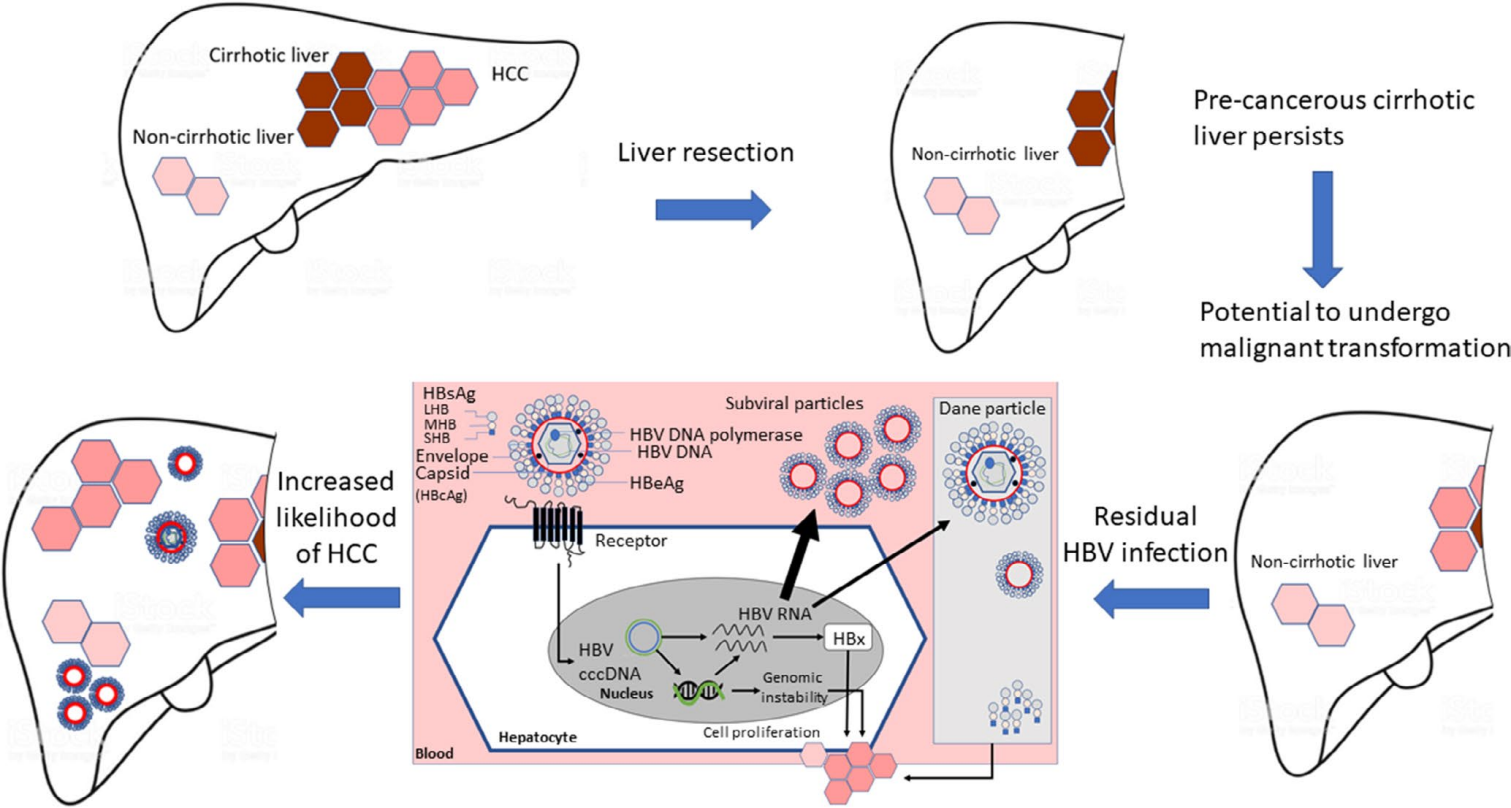
HCC recurrence after liver resection. After resection, cirrhotic liver retains the potential to undergo malignant transformation. This is possible in the absence of HBV‐derived oncogenic drivers but is increased in their presence. cccDNA, covalently closed circular DNA; HCC, hepatocellular carcinoma; HBV, hepatitis B virus; LHB, large HBV surface protein; MHB, medium HBV surface protein; SHB, small HBV surface protein; HBcAg, hepatitis B core antigen; HBeAg, hepatitis B ‘e’ antigen

Resection is also a preferred treatment option in patients with early‐stage HCC,^1^ and in Asia, although LT provides the best curative option, a relatively small donor pool means that resection is often the first‐line approach. Compared with rates of HCC recurrence post‐LT, recurrence rates post‐liver resection are much higher, at 56%‐70%.^50^, ^51^ Whereas it seems likely that the cause of HBV‐related HCC recurrence post‐LT is expansion of residual tumour cells containing integrated HBV DNA, after liver resection a different disease course is potentially more probable. After resection, cirrhotic liver, which is precancerous per se, will remain, with the potential to undergo malignant transformation regardless of the existence of HBV‐derived oncogenic drivers. The presence of residual HBV will further increase the likelihood of HCC recurrence, as with primary tumour formation (Figure 4). This risk may be largely that of late HCC recurrence^52^, ^53^ rather than early recurrence,^54^ with specific mutant antigens conferring a particularly high risk. The presence of pre‐S mutants in patients with HBV‐related HCC has been associated with a significantly higher risk of HCC recurrence after curative surgical resection.^55^ Of note, evaluation of predictive factors for HCC recurrence post curative resection for HBV‐related early‐stage HCC indicated that whereas preoperative HBV DNA levels ≥20 000 IU/mL were predictive of recurrence within 2 years (hazard ratio [HR] 2.77; *P* < .001), patients were at risk of late recurrence if preoperative HBsAg levels were ≥4000 IU/mL (HR 2.80; *P* = .023).^56^ Similar results were reported in patients receiving NAs after surgery; an HBsAg level of >200 IU/mL was associated with a 2‐fold increased likelihood of late recurrence (*P* = .027).^57^ Most recently, it has been shown that while an HBsAg level >200 IU/mL is an independent predictor for late recurrence (2‐5 years post‐resection), HBsAg levels >50 IU/mL could predict recurrence and mortality beyond 5 years. This suggests that different cut‐off values of HBsAg may be useful to predict outcomes at different intervals after surgical resection.^58^

Furthermore, in a prospective study of 89 patients undergoing liver resection for HBV‐related HCC, circulating HBsAg levels correlated with cccDNA copy number in both tumour and non‐neoplastic liver tissue but not with serum HBV DNA, total intrahepatic HBV DNA, viral replicative activity, or transcriptional activity. Correlation of HBsAg with cccDNA copy number, regardless of malignant status, suggests that a larger pool of cccDNA is associated with a higher rate of HBsAg production.^59^ It is important to distinguish the risk attributable to HBsAg positivity and HBV viraemia at the time of surgical intervention. Although risk of recurrence is high with HBV DNA levels ≥ 10^4^ copies/mL,^60^ high HBsAg levels despite low HBV DNA levels represent ongoing inflammation and thus are also associated with an increased risk of HCC recurrence.^61^ It may be expected that in patients with a high viral load, treatment with NAs will decrease HBV DNA levels, reduce liver inflammation resulting from HBV replication and therefore decrease the risk of HCC recurrence. This has been suggested in patients regardless of viral load^62^, ^63^ but remains to be proved unequivocally.

A large proportion of patients with HBV‐related HCC present late in their disease course, when potentially curative therapies are not an option, and there are currently no data on the association between HBsAg and advanced stages of HCC.

### 3.2 HBsAg in HCC: current and future implications for management

The Barcelona Clinic Liver Cancer (BCLC) staging system is one of several systems available. Although not a worldwide recommended categorization for HCC, it has been validated repeatedly and is recommended for prognostic prediction and treatment allocation.^1^ Management with curative intent, through resection or LT, is only possible in early stages of disease.^1^ Ablation is an option in patients with very early (stage 0) HCC, for the treatment of single, small nodules with a diameter of <2 cm, and in some patients with early (stage A) HCC who are not candidates for surgery.^1^

Involvement of HBsAg in the development of HCC has implications for its manipulation in the management of HBV‐related HCC in different settings, including first‐line therapy post‐LT and resection, even in the absence of reinfection. The observation that HBsAg has direct oncogenic potential suggests that targeting it may have therapeutic benefits. Hepatitis B immunoglobulin (HBIG) specifically targets HBsAg, acting both extracellularly in blood to bind and neutralize HBsAg in the envelope of circulating Dane particles and SVPs, and intracellularly, helping to prevent secretion of free HBsAg, active virions, and SVPs.^64^, ^65^, ^66^ Preliminary preclinical data showing that HBIG can initiate apoptosis in HCC tumour‐initiating stem‐like cells are promising (unpublished data), but supportive clinical data are required.

#### 3.2.1 Management post‐LT

Without prophylaxis, HBV infection returns in up to 75% of patients post‐LT,^67^ with risk of reinfection dependent on multiple factors, including HBsAg and HBV DNA positivity at the time of LT and presence of HCC.^64^, ^66^ Life‐long HBIG to mitigate this risk is well documented and can reduce reinfection risk to 19%‐36%,^67^, ^68^ and further still with concomitant NAs.^69^ Indeed, the concomitant use of third‐generation NAs and HBIG can decrease the rate of HBsAg persistence to <3% and is the current treatment standard. Patients with additional HBV DNA positivity at time of LT, patients with HCC, and patients with HBV/HDV coinfection are particularly prone to HBV reinfection without prophylaxis.^64^, ^66^, ^67^ Shorter courses of (lower dose) HBIG,^70^, ^71^ individualized dosing schedules,^72^, ^73^ HBIG limiting or sparing regimens,^74^ and/or de novo NAs alone^75^, ^76^ have also been evaluated to reduce the likelihood of HBV reactivation. A recent study has reported HBsAg loss in 100% (n = 41) of patients after 1 year of entecavir monotherapy post‐LT for HBV‐related HCC.^77^ However, one patient who experienced HCC recurrence in this study was also HBsAg positive after 33 months of HBsAg seroclearance, and robust data from controlled trials on the use of NAs alone are lacking regarding the control of HBsAg production,^78^ and current guidelines support the use of combination schedules.^1^ Indeed, a larger study has reported 15% of patients under entecavir monotherapy being HBsAg positive at 1 year post‐LT.^76^ HCC as an indication for LT remains a key risk factor for HBV reactivation^73^ and is associated strongly with the reappearance of HBsAg after withdrawal of HBIG.^75^ HBIGs are not indicated currently for the prevention of HCC recurrence post‐LT. However, the hypothesis that recurrence post‐LT is because of the expansion of residual tumour cells containing integrated HBV DNA, leading to both HCC recurrence and HBV reinfection, suggests that control of HBsAg is a reasonable therapeutic approach. In line with this, reduced risk of HCC recurrence post‐LT with prophylactic HBIG has been demonstrated.^79^ In this study, HCC recurrence was assessed in viraemic HBeAg‐positive patients given either low‐ or high‐dose HBIG. In patients satisfying the Milan criteria, the 3‐year rate of HCC recurrence‐free survival with high‐dose immunoglobulin was 97.2% compared with 83.2% with low‐dose HBIG (*P* = .042). In addition, a large retrospective analysis has suggested that combination therapy with immunoglobulin and NAs may reduce the rate of HCC recurrence. Although assessment of HCC recurrence was not the primary objective of the study, the reported annual incidence was very low at 1.7%^80^ and compares favourably with rates reported previously.^46^, ^76^

Although these studies are encouraging, it remains unclear whether the observations are because of reduced HBsAg directly, via immunomodulation properties of intravenous immunoglobulins and HBIG,^81^ or is a secondary benefit of the decreased inflammatory response generated by the presence of the virus. An additional possibility is that tumour‐initiating stem‐like cells (TICs) are circulating in the blood or that dormant TICs reside in other organs, including the lungs and the brain. LT for HCC does not always remove these TICs. After therapeutic pressure is lifted, proliferation of these dormant TICs will resume. Prophylaxis with HBIGs may not only remove circulating tumour cells (CTCs) but also dormant tissue‐resident CTCs. Immunotherapy is durable but not robust, while chemotherapy is robust but less durable. Surgical removal with prophylactic HBIG may not only remove TICs and CTCs but also eliminate residual dormant tumour cells. Therefore, this combination needs to be considered as the gold standard in LT.

Hepatitis B immunoglobulin and NAs may have less control over the replication of HBV within tumour cells compared with non‐cancerous cells, and a proportion of HBsAg produced by hepatocytes may be unaffected by the administration of NAs. In support of this, there is some evidence that HCC recurrence rates in post‐transplant patients with HBV DNA positivity are similar when treated with NAs alone or concomitantly with both HBIG plus NAs, whereas patients with detectable HBsAg respond much better when treated with combination therapy.

#### 3.2.2 Management post‐liver resection

As with liver transplant, HBIGs are not indicated currently for the prevention of HCC recurrence post‐liver resection, and their potential to prevent HCC recurrence in this setting is more doubtful than for following LT. Although low post‐resection levels of HBsAg may confer a survival benefit,^82^ there are currently no data on the implications of targeting HBsAg on HCC recurrence after resection.

The picture is complicated by challenges with comparing and interpreting data on the relative value of different prophylactic schedules. Patients undergoing LT or resection are a heterogeneous patient population, subject to different treatment protocols, and data available are largely from case studies and real‐world experience. What is really required to ascertain the potential role of HBsAg in the management of HCC recurrence post‐LT and resection is a trial comparing results with NAs plus HBIG vs NAs alone. Prevention strategies for the development of HCC in patients with HBV are of the utmost importance. Accordingly, there is a need to investigate further the role of HBsAg in mechanisms underlying HCC recurrence and to evaluate the impact of neutralizing HBsAg, most likely with HBIGs, as one crucial action for HCC prevention.

The current guidelines from the European Association for the Study of the Liver highlight the need for more therapeutic options for patients in the later stages of HCC.^1^ The future standard of care is likely to include immune‐oncology (IO) combinations, and although little is known about the role of HBsAg in the later stages of HCC, targeting this marker in combination with IOs in development with no rationale for use in HBV‐related HCC could be of benefit. To date, the role of therapeutic HBV vaccination in the HCC setting has not been demonstrated.

## 4 CONCLUSIONS

The role of HBsAg in the development of HCC and its recurrence post‐LT or resection has multiple implications for therapy. Preliminary data suggest that targeting HBsAg in primary disease could have an anti‐oncogenic effect, but there is a long way to go before anti‐HBsAg therapies will have a clinical application in this setting. Although precise pathways are yet to be elucidated, it seems probable that HBsAg is key in the recurrence of HCC after LT, although its role in HCC recurrence post‐resection is less clear. The role of HBsAg in HCC recurrence post‐LT warrants further investigation and suggests a potential role for immunotherapy in its future management. Given that HBIGs target HBsAg directly and that clinical experience over decades has shown them to be well tolerated, they may be a relevant combination partner in this setting.

## CONFLICT OF INTEREST

Authors disclose the following potential conflict of interest in the last 5 years. Related to manuscript topic: PS, PB, R‐HH, CMH, CL, KM, AV, DS; Biotest AG. Unrelated to manuscript topic: PS: Allergosan, ArgosMed, Astellas, Bard Medical, Baxter, Chiesi, Dr Fanz Köhler Chemie, Ethicon, Hexal, Medtronic, Merck, Neovii, Novartis, Panacea Biotec, Sandoz, Sanofi‐Aventis GmbH, Shire, TEVA‐ratiopharm; PB: Biotest, Kedrion, Chiesi Farmaceutici; R‐HH: No conflict of interest declared; CMH: Employee of Biotest AG; CL: Astellas, Novartis, Grand Fontaine, MSD, Ethicon, Medtronic, Roche; KM: Biotest AG; AV: AstraZeneca, Bayer, Bristol‐Myers Squibb, Eisai, Eli Lilly, Incyte Corporation, Ipsen, Merck, MSD, Pierre Fabre, Roche, Sanofi; DS: Astellas, Go liver, Gilead Sciences.

## PERMISSION TO REPRODUCE MATERIAL FROM OTHER SOURCES

No material has been reproduced from other sources. The figures generated are original.

## Data Availability

Data sharing is not applicable to this article as no new data were created or analysed in this study.
